# Molecular Mechanisms Underlying Cucumber Resistance to Cucumber Green Mottle Mosaic Virus: From Host–Virus Interactions to Precision Breeding Strategies

**DOI:** 10.3390/ijms27198569

**Published:** 2026-09-25

**Authors:** Xiaoting Yu, Yuqiang Zhu, Xin Wang, Yuyan Sun, Luyao Gao, Shengjun Zhou, Ming Xin, Peng Zhang

**Affiliations:** 1 College of Horticulture, Northeast Agricultural University, Harbin 150030, China; 2 Zhejiang Academy of Agricultural Sciences, Hangzhou 310021, China

**Keywords:** cucumber green mottle mosaic virus, CRISPR/Cas genome editing, host–virus interaction, resistance gene, marker-assisted selection, precision breeding

## Abstract

Cucumber (*Cucumis sativus* L.) is an economically important vegetable crop cultivated worldwide, but its sustainable production is increasingly threatened by Cucumber green mottle mosaic virus (CGMMV). CGMMV is a positive-sense single-stranded RNA virus belonging to the genus *Tobamovirus* and represents a major constraint on cucurbit production because of its efficient seed transmission, mechanical spread, environmental persistence, and broad host range. Infection can cause leaf mosaic and mottling, growth suppression, and severe fruit symptoms, including internal discoloration, water-soaked tissues, and flesh deterioration, resulting in substantial losses in yield and commercial quality. Recent advances in cucumber genomics, resistance-germplasm screening, host–virus interaction studies, and molecular breeding have provided new opportunities for improving CGMMV resistance. However, progress remains constrained by the scarcity of well-characterized resistance donors, the limited functional validation of cucumber-specific resistance genes, isolate-dependent resistance responses, and the relatively low efficiency of cucumber transformation and genome editing. This review summarizes current knowledge of CGMMV infection processes, host susceptibility factors, antiviral defense pathways, resistance resources, and resistance-associated loci in cucumber and related cucurbits. Evidence is interpreted according to host species and the level of experimental validation. It further evaluates the potential of marker-assisted selection, genomic selection, genome editing, and RNA-based engineering and outlines an integrated framework for translating molecular findings into durable CGMMV-resistant cucumber cultivars.

## 1. Introduction

Cucumber green mottle mosaic virus (CGMMV), a positive-sense single-stranded RNA virus belonging to the genus *Tobamovirus* within the family *Virgaviridae*, is one of the most destructive viral pathogens affecting cucumber and other cucurbit crops worldwide. CGMMV was first described in cucumber in England in 1935 [1]. Its epidemiological persistence is reinforced by seed-associated inoculum that may remain infective after commonly used chemical and heat disinfection treatments [2,3,4,5], prolonged survival in infested soils [6], and susceptible weed hosts that may act as potential virus reservoirs [7]. Early infection can substantially reduce cucumber yield [8], whereas infected cucumber plants may occasionally remain symptomless [9]. Unlike many foliar-transmitted viruses, CGMMV exhibits strong persistence and epidemiological complexity due to its efficient seed transmission, mechanical transmission, and survival in contaminated production environments [10,11,12,13,14]. Field and greenhouse studies have further documented transmission associated with pollen and pollination, plant-to-plant contact, and contaminated production inputs [3,4,5,11,12,13,14]. Since its emergence in major cucurbit-producing regions, CGMMV outbreaks have caused severe economic losses by inducing leaf mosaic symptoms, fruit deterioration, and reductions in commercial quality [10,15,16,17]. Recent surveillance studies have documented regional occurrence and genetic variation of CGMMV, including Asian and European virus populations, while broader cucurbit-virus surveys provide additional production-context information [18,19,20,21,22,23,24,25,26,27,28,29,30,31]. Although quarantine, seed disinfection, and cultivation management practices have been widely adopted to reduce virus spread, these strategies cannot provide inheritable resistance within cucumber plants. Therefore, developing CGMMV-resistant cucumber cultivars through genetic improvement has become an essential approach for achieving sustainable disease management.

Achieving effective genetic resistance against CGMMV requires a comprehensive understanding of the molecular interactions between cucumber and the virus, as well as the genetic determinants controlling resistance. Current management strategies, including seed sanitation, quarantine measures, and hygienic practices, mainly aim to reduce virus introduction and transmission, but they cannot provide genetic resistance once plants are exposed to the pathogen [10,15]. Elucidating cucumber-CGMMV interactions can facilitate the identification of host factors involved in viral replication, movement, and defense regulation, thereby providing potential targets for resistance improvement. Furthermore, characterization of resistance mechanisms can advance understanding of cucumber antiviral defense and provide a basis for identifying resistance genes and improving germplasm. Meanwhile, advances in molecular breeding technologies, including marker-assisted selection and genome editing, provide new opportunities to accelerate the development of CGMMV-resistant cucumber cultivars by improving the efficiency and precision of resistance selection [32,33,34,35,36,37].

This review summarizes recent advances in CGMMV resistance research in cucumber, focusing on host–virus interactions, genetic determinants, resistance mechanisms, and molecular breeding strategies. Evidence obtained in cucumber is distinguished from evidence derived from related cucurbits and model plants, and each finding is interpreted according to its host species and level of experimental validation. Current challenges and future perspectives for integrating resistance-gene discovery with emerging breeding technologies are also considered in relation to the development of durable CGMMV-resistant cucumber cultivars.

To make the evidence boundaries explicit, the studies discussed in this review are classified according to host species, experimental validation, and breeding relevance (Table 1).

## 2. Viral Infection Processes and Pathogenic Determinants

### 2.1. Overview of CGMMV Infection

Following entry into damaged host tissues, CGMMV infection involves viral genome replication, cell-to-cell movement, and subsequent systemic spread, which collectively determine symptom development and disease severity in cucumber [10]. The movement and systemic phases also depend on the viral movement protein and host transport processes [54,55]. Viral RNA replication is generally associated with membrane-bound replication complexes containing viral replication proteins and host cellular factors. However, much of the current understanding of these structures is derived from model tobamoviruses, and the detailed organization of CGMMV replication complexes remains insufficiently characterized [51,56]. The CGMMV genome retains the canonical tobamovirus organization. Additional small proteins have recently been reported [57], a symptom-attenuating single-amino-acid substitution in the intervening region of the 129K protein has been characterized [58], and the 54-kDa and 4–6-kDa products have been reviewed in the broader tobamovirus literature [59]. These viral determinants may influence pathogenicity, but they do not by themselves establish a cucumber resistance gene. In the movement stage, the viral movement protein (MP) promotes transport through plasmodesmata, while the interaction between CGMMV MP and coat protein (CP) contributes to efficient systemic infection [60]. Therefore, viral replication and movement processes represent important mechanistic entry points for identifying host susceptibility and resistance-associated factors. The proposed infection process and major host defense-associated pathways are summarized in Figure 1. The host-level disease cycle and associated phenotypes are summarized in Figure 2.

The schematic summarizes CGMMV replication, movement, systemic infection, and major host defense pathways, including RNA silencing, hormone signaling, ROS responses, and the autophagy-NMD relationship. Autophagy is shown as an upstream regulator of NMD. The figure was redrawn based on comparative tobamovirus and CGMMV studies [42,43,44,45,48,51,52,53,56,60,61,62,63,64,65,66,67,68,69,70,71,72,73,74,75,76,77,78]. The scientific content was prepared and verified by the authors; OpenAI Codex (GPT-5) was used only for layout, vector drawing, and typesetting. The figure is schematic, not to scale, and contains no original experimental data.

CGMMV has a positive-sense single-stranded RNA genome of approximately 6.4 kb and retains the canonical tobamovirus coding strategy. The genomic RNA produces two replication-associated proteins, the 129-kDa protein and the 186-kDa readthrough product, whereas subgenomic RNAs express the 29-kDa movement protein and the approximately 17.3–17.4-kDa coat protein [51,56,57,58,59,60]. The 129K/186K replication module is relevant to genome amplification and the virus-host RNA-silencing interface, while MP and CP contribute to cell-to-cell movement, encapsidation, and systemic infection [51,60]. This functional division provides the molecular framework for interpreting the viral determinants and host-response evidence discussed below.

Recent work indicates that the canonical four-protein scheme does not capture all CGMMV coding potential. Gong et al. detected rORF1- and rORF2-derived peptides by mass spectrometry and supported their translation by ribosome profiling; these additional ORFs are located on the negative-sense RNA, and the authors proposed an internal-ribosome-entry-site-mediated translation mechanism. Mutation of either rORF reduced virulence, whereas ectopic expression could rescue the pathogenicity of the mutants [57]. These results support rORF1/rORF2 as newly characterized virulence-associated products. However, their host targets, relative contributions to replication versus symptom development, and conservation across CGMMV isolates remain insufficiently resolved; they should not yet be described as validated cucumber resistance determinants.

In addition, the broader tobamovirus literature describes a 54-kDa product and 4–6-kDa proteins beyond the canonical ORFs. The 54-kDa product has been discussed as a putative, translation-associated product related to the replicase/RdRp region, whereas the functions of the 4–6-kDa proteins remain poorly defined [59]. Because the available source is a comparative review and does not establish a complete CGMMV-specific function for these products, they should be treated as unresolved coding/functional hypotheses rather than validated cucumber resistance determinants or established CGMMV RNA-silencing suppressors.

#### 2.1.1. Movement Protein as a Determinant of CGMMV Movement and Host Interaction

Movement protein is a major viral determinant of cell-to-cell movement and systemic infection. Like other members of the 30-kDa movement-protein superfamily, tobamovirus MP binds viral RNA, targets plasmodesmata, and modifies their permeability to facilitate intercellular transport [61,63]. In Tobacco mosaic virus, MP activity can also be regulated by host-associated phosphorylation processes [64]. In CGMMV, MP also interacts directly with CP, and disruption of the MP-CP interaction reduces viral systemic accumulation, demonstrating that coordinated functions of these two viral proteins contribute to long-distance infection [60].

MP-mediated movement also depends on interactions with host proteins. In Tobacco mosaic virus, pectin methylesterase interacts with MP and participates in viral cell-to-cell movement [62]. This provides a comparative model for identifying plasmodesmata-associated host factors in CGMMV, although an equivalent interaction has not been functionally established in cucumber. A cucumber small heat-shock protein has been reported to interact with the CGMMV helicase domain, but its contribution to resistance remains unresolved [79]. WPRb-associated evidence is species- and system-dependent: in watermelon, *ClWPRb* variation was associated with recessive resistance and an allele-specific marker was validated in a backcross population; in heterologous *Nicotiana benthamiana* assays, *ClWPRb* localized to plasmodesmata and interacted with CGMMV MP, while disruption of the endogenous WPRb homolog restricted systemic spread without eliminating protoplast-level viral accumulation [43]. Thus, WPRb is a well-supported watermelon resistance-associated host factor with functional evidence from heterologous assays, but the cucumber ortholog remains unvalidated.

#### 2.1.2. *Tobamovirus* Replication Complexes and Host Susceptibility Factors

CGMMV RNA replication is expected to occur in virus-induced, membrane-associated replication complexes. In model tobamoviruses, replication proteins recruit and remodel endoplasmic-reticulum membranes, creating a specialized environment for viral RNA synthesis [51,56]. For CGMMV, the existence of a comparable replication framework is supported by its conserved replicase organization, but the ultrastructure, composition, and host-factor requirements of CGMMV replication complexes have not been resolved at the same level. This distinction is important: conserved genome organization supports a mechanistic analogy, whereas direct CGMMV evidence for individual replication-complex components remains limited [51,60].

RNA silencing is a central host barrier to positive-sense RNA viruses. Viral replication generates double-stranded RNA substrates that can initiate antiviral silencing, whereas viral suppressors interfere with this defense [52,67,68,69]. The CGMMV 129-kDa protein has experimentally supported RNA-silencing suppressor activity [58].

Studies of model tobamoviruses have identified members of the *Tobamovirus* Multiplication (TOM) family as host susceptibility factors required for efficient viral replication. Simultaneous RNA interference-mediated suppression of *NtTOM1* and *NtTOM3* nearly abolished tobamovirus multiplication in tobacco [49]. Similarly, simultaneous mutations in homologous TOM-family genes strongly restricted tobamovirus multiplication in *Arabidopsis* [50]. These findings identify TOM-family proteins as conserved host susceptibility factors and provide a mechanistic basis for investigating cucumber TOM homologs as potential CGMMV susceptibility factors. However, no cucumber TOM-family gene has yet been functionally validated as a CGMMV resistance target; consequently, their application in cucumber genome editing remains a future research direction. Infectious CGMMV clones and CGMMV-derived vectors have also been developed to support functional analysis of viral and host determinants [80,81].The principal viral determinants and host-interaction evidence discussed in this section are summarized in Table 2.

**Table 2:** CGMMV viral determinants and host-interaction evidence.

Viral Determinant or Process	Core Observation or Function	Host/System	Evidence Level	Key Literature (Journal)
Genome organization and replication proteins	CGMMV is a positive-sense RNA tobamovirus with a canonical replicase-movement protein-coat protein organization; tobamovirus RNA replication is associated with membrane-linked replication complexes.	CGMMV and comparative tobamovirus systems	Genomic and comparative mechanistic evidence	Shi et al. (2023), Molecular Plant Pathology [60]; Ishibashi and Ishikawa (2016), Annual Review of Phytopathology [51]; Más and Beachy (1999), Journal of Cell Biology [56]
129-kDa protein and intervening region	A single amino-acid substitution in the 129-kDa protein is associated with attenuated symptoms and altered RNA-silencing-suppressor-related activity.	CGMMV infection assays	Direct CGMMV functional evidence	Chen et al. (2020), Phytopathology [58]
Additional rORF1 and rORF2 proteins	Small proteins translated from the negative-strand RNA through an internal ribosome entry site contribute to virulence; mutation or ectopic-expression assays support specific functions.	CGMMV and infected host plants	Direct CGMMV functional evidence	Gong et al. (2025), Science China Life Sciences [57]
54-kDa and 4–6-kDa proteins	These less-characterized tobamovirus proteins have distinct subcellular localizations and possible virulence-related functions.	CGMMV and comparative tobamoviruses	CGMMV plus comparative synthesis	Palukaitis et al. (2024), Viruses [59]
MP-CP interaction	Interaction between the CGMMV movement protein and coat protein promotes systemic infection; disruption reduces systemic accumulation.	CGMMV	Direct protein-interaction and infection assays	Shi et al. (2023), Molecular Plant Pathology [60]
WPRb host factor and plasmodesmata-mediated movement	In watermelon, the *ClWPRb* G1282A/V428I variant was associated with recessive resistance and an allele-specific marker was validated in a backcross population. Localization, MP-interaction, protoplast, and whole-plant assays in heterologous systems linked WPRb to CGMMV movement and systemic spread.	Watermelon genetic populations; heterologous *Nicotiana benthamiana* and protoplast assays	Direct watermelon genetic/marker evidence plus heterologous functional evidence; cucumber ortholog unvalidated	Cai et al. (2023), Plant Physiology [43]
Movement protein and plasmodesmata	*Tobamovirus* movement proteins bind viral RNA and alter plasmodesmatal permeability to promote cell-to-cell trafficking.	Tobacco mosaic virus and comparative tobamovirus systems	Comparative mechanistic evidence	Wolf et al. (1989), Science [61]; Lucas (2006), Virology [63]
Movement-protein host regulation	Pectin methylesterase interaction and phosphorylation regulate movement-protein-associated trafficking in tobacco mosaic virus.	Tobacco mosaic virus in tobacco	Comparative mechanistic evidence	Chen et al. (2000), EMBO Journal [62]; Waigmann et al. (2000), EMBO Journal [64]
sHSP-helicase interaction	A small heat-shock protein interacts with the CGMMV helicase domain; its contribution to resistance remains unresolved.	CGMMV host system	Interaction evidence; resistance function incomplete	Liu et al. (2019), Phytopathology [79]
TOM-family susceptibility factors	*NtTOM1*/*NtTOM3* suppression or homologous TOM mutations strongly restrict tobamovirus multiplication.	Tobacco and *Arabidopsis*; model tobamoviruses	Comparative host-factor evidence	Asano et al. (2005), FEBS Letters [49]; Yamanaka et al. (2002), Journal of Virology [50]
Infectious clones and viral vectors	Full-length infectious clones and a CGMMV-derived VIGS vector enable reverse genetics and host-factor testing.	CGMMV in cucurbit systems	Experimental tools	Park et al. (2017), Virus Genes [80]; Liu et al. (2020), Plant Methods [81]

### 2.2. Genetic Architecture of CGMMV Resistance in Cucurbit Crops

The genetic architecture of CGMMV resistance in cucurbit crops remains incompletely understood, and current evidence does not support a single conserved inheritance model. Most evaluated *C. sativus* accessions were susceptible or exhibited only partial resistance, and responses differed between European and Asian CGMMV strains [31,38]. However, the causal loci and inheritance mechanisms underlying these phenotypes remain insufficiently characterized.

Studies in related cucurbit crops further demonstrate that CGMMV resistance is genetically diverse rather than controlled by a universally conserved mechanism. In muskmelon, early genetic studies suggested that resistance was controlled by recessive polygenes rather than by a single major gene [82]. In watermelon, WPRb was identified as a host factor recessively associated with CGMMV resistance through regulation of plasmodesmata-mediated viral movement [43]. These findings provide valuable comparative evidence for understanding CGMMV resistance mechanisms in cucurbit crops. However, resistance-related genes identified in melon and watermelon cannot be directly regarded as validated resistance genes in cucumber because of differences in genetic background, virus isolate response, and host-virus interactions. Therefore, identifying cucumber-specific resistance loci and clarifying their functional mechanisms remain essential objectives for future resistance breeding.

#### WPRb-Associated Resistance in Watermelon: Genetic and Heterologous Functional Evidence

Among the resistance mechanisms identified in cucurbit crops, WPRb-associated resistance is a well-characterized example of host-factor-mediated restriction of CGMMV movement. The evidence comes from two complementary levels: genetic mapping and marker validation in watermelon, and molecular and functional assays performed in heterologous systems, mainly *Nicotiana benthamiana*. WPRb should therefore be interpreted as a watermelon resistance-associated host factor and a comparative model for cucumber research, rather than as direct evidence for a validated cucumber resistance gene [43].

In watermelon, a segregating population derived from the CGMMV-tolerant accession PI595203 and the susceptible line M1511-3 was used for genetic analysis. F_1_ progeny were susceptible, consistent with a recessive component, whereas the F_2_ segregation did not fit the expected 1:3 ratio for a single recessive gene, suggesting additional genetic factors. QTL-seq identified a resistance-associated region on chromosome 4, within which a nonsynonymous G1282A substitution in *ClWPRb* caused a V428I amino acid change and was strongly associated with the resistance phenotype. An allele-specific marker based on this variant was validated in a watermelon backcross population, supporting its use for tracking the resistance-associated allele in watermelon breeding [43].

The mechanistic experiments were not all performed in watermelon. In heterologous *Nicotiana benthamiana* systems, *ClWPRb* localized to plasmodesmata and interacted with CGMMV MP in BiFC and co-immunoprecipitation assays. Loss of WPRb function did not significantly reduce virus accumulation in protoplast assays, but delayed systemic infection and reduced viral accumulation in whole plants. WPRb disruption was also associated with increased callose deposition at plasmodesmata and reduced intercellular transport. Because edited plants eventually developed symptoms, these experiments support enhanced tolerance or delayed disease progression rather than complete immunity [43].

From a breeding perspective, the G1282A-associated marker is a watermelon marker for tracking the resistance-associated WPRb allele; it should not be described as a validated cucumber marker. The durability of WPRb-associated resistance against genetically diverse CGMMV isolates, its performance under field conditions, and possible effects on plant growth and fruit quality require further investigation. More importantly, the cucumber WPRb ortholog has not yet been functionally validated for CGMMV resistance. WPRb should therefore be regarded as a cross-species mechanistic reference and a candidate cucumber susceptibility factor, rather than an established cucumber resistance gene. Future studies should identify and test cucumber WPRb homologs using genetic analysis, molecular interaction assays, complementation or loss-of-function approaches, and multi-isolate resistance evaluation [43].

### 2.3. Molecular Mechanisms of Cucumber Resistance to CGMMV

The molecular basis of cucumber resistance to CGMMV has not yet been fully elucidated and cannot be attributed to a single defense pathway. Available evidence from cucumber and related experimental systems suggests that antiviral responses involve multiple regulatory layers, including host–virus protein interactions, RNA silencing, phytohormone signaling, and cellular RNA-quality-control pathways. CGMMV-responsive miRNAs have been identified in cucumber, although their direct antiviral functions remain unverified [83]. Functional evidence for ClAGO1- and ClAGO5-mediated antiviral defense and for the ClWRKY70-ClACO5 regulatory module has mainly been obtained in watermelon [44,45], whereas the interaction between nonsense-mediated mRNA decay and autophagy has primarily been characterized in *Nicotiana benthamiana* [48]. Therefore, these mechanisms should be interpreted according to their host species and level of functional validation rather than regarded as a fully established cucumber-specific regulatory network. General antiviral-defense studies describe interconnected RNA-silencing, hormone, autophagy, and RNA-quality-control pathways, but these frameworks do not substitute for CGMMV-specific cucumber validation [52,53,67,68,69,70,71,72,73]. Recent cucumber-specific experiments further implicated fatty-acid metabolism: CGMMV altered fatty-acid composition and *CsSSI2* expression or localization, whereas *CsSSI2* silencing reduced viral RNA and coat-protein accumulation. This identifies a cucumber-supported host process, but not yet a naturally segregating resistance locus [42]. CGMMV-associated DNA methylation and m6A changes have also been described in watermelon [84,85]; these observations broaden candidate regulatory layers but do not establish heritable resistance in cucumber. In bottle gourd and watermelon, CGMMV infection was associated with chlorophyllase 1 induction and chlorophyll changes, whereas the chlorophyllase response was not observed in *N. benthamiana*, illustrating host dependence [66]. A CGMMV coat-protein study reported associations with host proviral factors, condensate formation, and photosynthesis remodeling, while a separate study linked CGMMV coat protein to mitochondrial ATPδ and ROS in *N. benthamiana*. These findings are mechanistically informative but do not constitute cucumber-specific resistance validation [74,75]. The relationships among these defense layers and their evidence boundaries are summarized in Figure 3.The host systems, evidence strengths, and validation boundaries for these defense pathways are summarized in Table 3.

**Figure 3:**
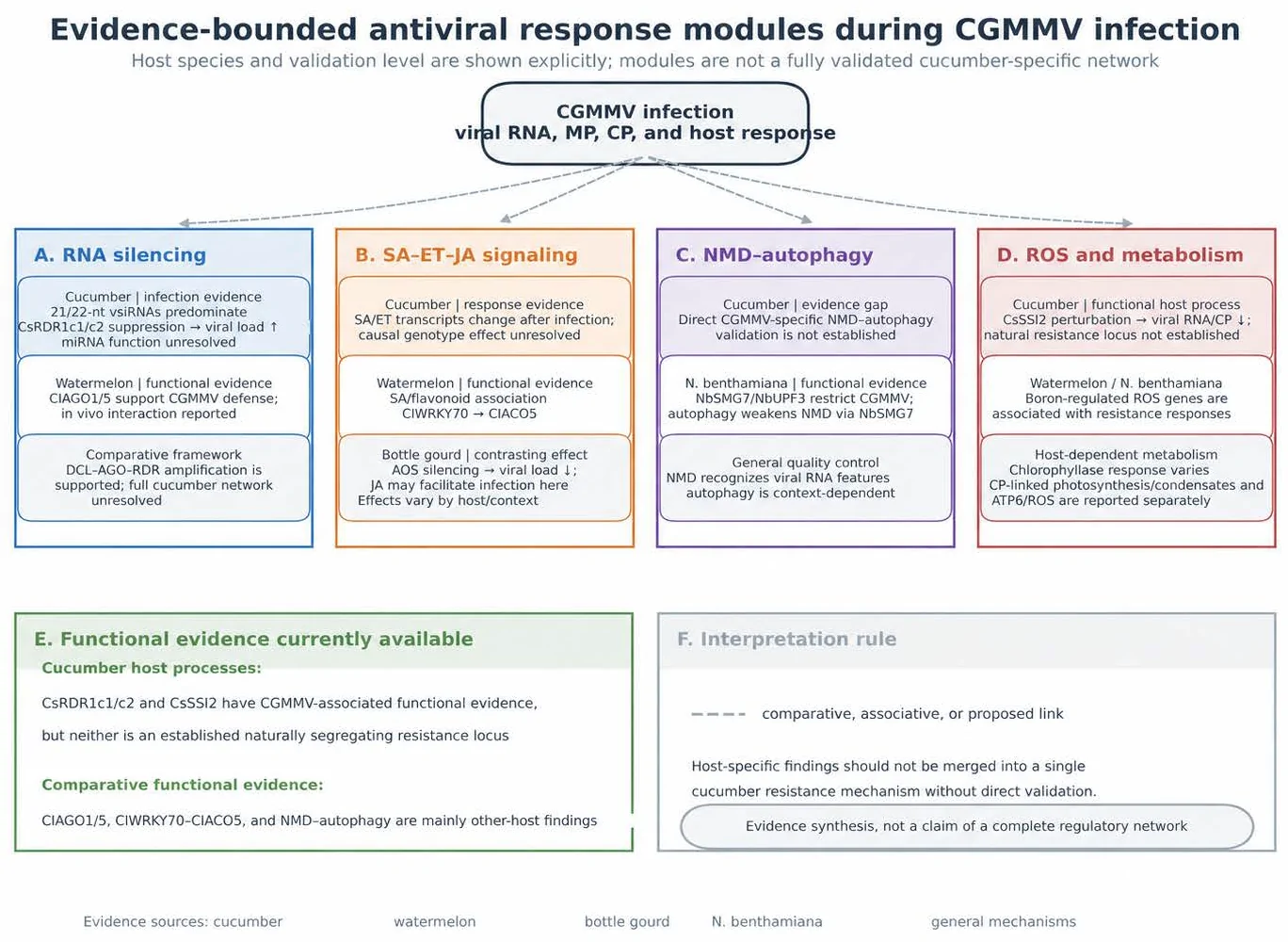
Antiviral response modules associated with CGMMV infection. The schematic summarizes RNA silencing, hormone signaling, NMD-autophagy, and ROS/metabolic responses reported in cucumber, watermelon, bottle gourd, and *Nicotiana benthamiana*. The figure distinguishes infection-associated observations from experimentally supported functional relationships and does not represent a fully validated cucumber-specific regulatory network. The figure was redrawn based on the cited literature [40,42,44,45,46,48,52,53,65,66,67,68,69,70,71,72,73,74,75,76,77,78,83,86,87,88,89,90]. The scientific content was prepared and verified by the authors; OpenAI Codex (GPT-5) was used only for layout, vector drawing, and typesetting. The figure contains no original experimental data.

**Table 3:** CGMMV-responsive defense pathways and validation boundaries.

Pathway or Process	Gene, Marker, or Observation	Host	Evidence Strength and Boundary	Key Literature (Journal)
Antiviral RNA silencing	21- and 22-nt CGMMV-derived siRNAs predominate in infected plants; this supports activation of antiviral silencing but does not define a complete DCL-AGO-RDR network.	Cucumber	Infection-associated molecular evidence; network incomplete	Li et al. (2016), Archives of Virology [87]; Baulcombe (2004), Nature [67]; Brodersen and Voinnet (2006), Trends in Genetics [68]; Ding and Voinnet (2007), Cell [86]
CGMMV-responsive miRNAs	Novel and conserved cucumber miRNAs change after infection; direct antiviral functions remain unverified.	Cucumber	Profiling evidence only	Liu et al. (2015), PLOS ONE [83]
*CsRDR1*-mediated defense	Suppression of *CsRDR1c1*/*c2* increases viral accumulation, supporting a contribution to antiviral defense.	Cucumber	Functional evidence; natural resistance allele not established	Leibman et al. (2018), Molecular Plant Pathology [40]
AGO-mediated defense	*ClAGO1* and *ClAGO5* are induced by infection and positively contribute to defense; in vivo interaction was reported.	Watermelon	Functional comparative evidence; cucumber homologs remain unvalidated	Liu et al. (2023), Molecular Plant Pathology [44]
Hormone-responsive transcription	SA- and ethylene-related genes are differentially expressed during CGMMV infection, but genotype-dependent resistance effects are not established.	Cucumber	Transcriptomic evidence; pathway causality unresolved	Slavokhotova et al. (2021), Life [88]; Zhao and Li (2021), PLOS Pathogens [71]
SA and flavonoid metabolism	Resistant watermelon shows SA-associated transcriptional and metabolic changes; SA treatment and flavonoid-associated remodeling correlate with lower viral accumulation.	Watermelon	Integrative and functional comparative evidence	Liu et al. (2023), Journal of Experimental Botany [89]
Ethylene-*ClWRKY70*-*ClACO5* module	*ClACO5* silencing increases susceptibility; ClWRKY70 binds the *ClACO5* promoter and activates ethylene biosynthesis; heterologous expression enhances resistance.	Watermelon and *Nicotiana benthamiana*	Functional comparative evidence; cucumber transfer not established	Liu et al. (2024), Frontiers in Plant Science [45]
Jasmonic-acid signaling	*AOS* silencing reduces viral accumulation, suggesting that JA signaling may facilitate infection in bottle gourd.	Bottle gourd	Host-dependent functional evidence	Li et al. (2023), International Journal of Molecular Sciences [46]; Li et al. (2019), International Journal of Molecular Sciences [90]
NMD-autophagy crosstalk	NbSMG7 and NbUPF3 restrict CGMMV; autophagy-dependent NbSMG7 degradation weakens NMD-mediated restriction.	*Nicotiana benthamiana*	Direct CGMMV functional evidence; not demonstrated in cucumber	Chen et al. (2024), Advanced Science [48]; Garcia et al. (2014), Cell Host & Microbe [76]; Chen et al. (2024), New Plant Protection [77]; Yang and Liu (2022), FEBS Letters [78]
ROS and boron response	ROS-related genes participate in boron-regulated responses to CGMMV infection.	*Nicotiana benthamiana* and watermelon	Functional comparative evidence	Guo et al. (2022), Frontiers in Plant Science [65]
Chlorophyll catabolism	CGMMV decreases chlorophyll a and upregulates Chlorophyllase 1 in cucurbit crops.	Cucurbit crops	Infection-associated metabolic evidence; causal resistance role unresolved	Li et al. (2025), Plants [66]
Fatty-acid metabolism and *CsSSI2*	Modulation of fatty-acid metabolism and *CsSSI2*-associated responses contributes to reduced CGMMV infection in cucumber.	Cucumber	Recent cucumber functional evidence	Jiang et al. (2025), Plant Cell Physiology [42]
CP-associated condensates and photosynthesis	CGMMV coat protein hijacks pro-viral factors to generate condensates and remodel photosynthesis.	*Nicotiana benthamiana*	CP-condensate/NbFBPase assays in *N. benthamiana*; cucumber resistance function remains unvalidated	Yin et al. (2025), Science Bulletin [74]
Mitochondrial ATPδ	CGMMV coat protein hijacks mitochondrial ATPδ to promote infection.	*Nicotiana benthamiana*	Interaction, silencing, and overexpression evidence in *N. benthamiana*; no cucumber resistance allele established	Yang et al. (2024), Molecular Plant Pathology [75]

#### 2.3.1. RNA Silencing as a Conserved Antiviral Defense Pathway

RNA-dependent RNA polymerases (RDRs) further amplify antiviral silencing by generating secondary double-stranded RNA substrates, thereby strengthening antiviral responses [86]. The broader antiviral RNA-silencing literature supports this amplification model, although the specific CGMMV-cucumber DCL-AGO-RDR network remains incompletely defined [52,67,68,69]. Deep sequencing of CGMMV-infected cucumber plants revealed that 21- and 22-nt virus-derived small interfering RNAs were the predominant size classes, supporting the activation of antiviral RNA silencing during infection [87]. High-throughput sequencing further identified multiple conserved and novel cucumber miRNAs responsive to CGMMV infection, indicating extensive post-transcriptional regulatory changes in the cucumber-CGMMV interaction. However, the direct antiviral functions of these miRNAs have not yet been experimentally established [83].

However, direct evidence defining the complete DCL-AGO-RDR regulatory network during CGMMV infection in cucumber remains limited. The most detailed functional evidence has been obtained from watermelon. Comparative genomic and transcriptomic analyses identified multiple AGO, DCL, and RDR family members responsive to CGMMV infection. Among these components, *ClAGO1* and *ClAGO5* exhibited strong transcriptional induction after infection, and functional analyses demonstrated that both genes positively contribute to watermelon defense against CGMMV, while protein-interaction assays indicated that ClAGO1 and ClAGO5 interact in vivo [44].

Further analysis revealed potential links between ethylene signaling and RNA silencing regulation. Promoter analysis identified ethylene response factor (ERF)-binding elements in *ClAGO1* and *ClAGO5*, and exogenous ethylene treatment increased *ClAGO5* expression. Several ERF genes also showed co-expression patterns with *ClAGO5*, suggesting that ethylene may enhance antiviral RNA silencing through transcriptional regulation of AGO genes. Nevertheless, direct binding and transcriptional activation of *ClAGO5* by specific ERFs require further experimental confirmation [44].

In cucumber, four *CsRDR1* genes exhibit distinct expression patterns during infection by several plant viruses, including CGMMV. Experimental suppression of *CsRDR1c1*/*c2* increased viral accumulation, supporting their contribution to antiviral defense [40]. However, it remains unknown whether natural allelic or expression variation in *CsRDR1* genes explains differences in CGMMV resistance among cucumber accessions. Therefore, RNA silencing should be regarded as an important component of CGMMV defense, while the specific genetic determinants responsible for resistance variation in cucumber require further investigation.

The CGMMV 129-kDa replication-associated protein also counteracts antiviral RNA silencing. Chen et al. showed that the 129-kDa protein and its methyltransferase domain plus intervening region from a severe CGMMV strain suppressed GFP-induced silencing and bound siRNAs. These activities were impaired in the corresponding proteins from an attenuated strain, linking the intervening-region substitution to altered silencing suppression and symptom attenuation [58]. These findings connect viral counter-defense with the host silencing responses summarized above.

#### 2.3.2. Phytohormone Signaling in CGMMV Responses: Roles of Salicylic Acid, Ethylene, and Jasmonic Acid

Phytohormone signaling participates in plant responses to CGMMV infection; however, the contribution of individual hormones varies among host species and should not yet be interpreted as a unified cucumber-specific regulatory network. Transcriptomic analysis of CGMMV-infected cucumber revealed substantial changes in hormone-related gene expression, particularly involving salicylic acid (SA) and ethylene (ET) signaling pathways. These transcriptional changes indicate that hormone-mediated regulation participates in cucumber responses to CGMMV infection, although direct evidence demonstrating that these pathways determine resistance differences among cucumber genotypes remains unavailable [88]. General hormone-immunity studies provide a comparative framework for interpreting SA and ET responses, but they do not establish a universal CGMMV resistance module [71,72,73].

More direct functional evidence has been obtained from watermelon. Comparative transcriptomic, metabolomic, and hormone analyses between CGMMV-resistant and susceptible watermelon materials showed that resistant plants exhibited enhanced expression of SA-associated genes and increased accumulation of SA-related metabolites. CGMMV infection further induced SA-responsive genes, including *PAL1*, *WRKY70*, and *PR1*. Exogenous SA treatment and flavonoid-associated metabolic regulation were also associated with reduced viral accumulation, suggesting that SA-mediated metabolic remodeling contributes to watermelon resistance against CGMMV [89].

Ethylene signaling has also been functionally linked to CGMMV resistance in watermelon. CGMMV infection induces ethylene production and increases the expression of *ClACO5*, a key gene involved in ethylene biosynthesis. Silencing of *ClACO5* increases susceptibility, whereas heterologous expression enhances resistance in *Nicotiana benthamiana*. Further analyses demonstrated that the SA-responsive transcription factor ClWRKY70 binds to the promoter region of *ClACO5* and activates its expression, establishing a positive regulatory relationship between SA-associated transcriptional regulation and ethylene biosynthesis during CGMMV defense [45].

By contrast, jasmonic acid (JA) signaling appears to exhibit host-dependent effects. In bottle gourd, integrated transcriptomic and metabolomic analyses showed that CGMMV infection activated JA biosynthesis and signaling. Silencing of the JA biosynthetic gene *AOS* reduced viral accumulation, suggesting that JA signaling may facilitate CGMMV infection in this host [46]. These findings indicate that SA, ET, and JA pathways should not be simply classified as synergistic antiviral mechanisms. Their functions depend on host genotype, infection stage, and virus–host interaction context. Future studies combining hormone quantification, genetic manipulation, and resistant versus susceptible cucumber comparisons are required to establish a cucumber-specific hormone regulatory model. More broadly, interactions among SA, ET, and JA signaling pathways may be synergistic or antagonistic depending on the host species, pathogen, developmental stage, and infection context [90]. Therefore, the three pathways should not be presented as a universally coordinated antiviral module during CGMMV infection.

#### 2.3.3. Crosstalk Between Nonsense-Mediated mRNA Decay and Autophagy During CGMMV Infection

Nonsense-mediated mRNA decay (NMD) is an RNA-surveillance pathway that eliminates aberrant transcripts and can also restrict plant RNA viruses carrying NMD-triggering features, such as internal termination codons or unusually long 3′ untranslated regions [76,77]. Autophagy represents another cellular quality-control pathway with both antiviral and proviral functions in plant-virus interactions [78]. However, direct evidence linking these two pathways during CGMMV infection has mainly been obtained in *Nicotiana benthamiana* rather than cucumber.

In *N. benthamiana*, CGMMV infection affected the expression and activity of core NMD factors, including NbSMG7 and NbUPF3. Overexpression of these factors reduced viral accumulation, whereas their suppression promoted infection, supporting a positive role for NMD in restricting CGMMV [48]. Viral RNA features recognized by the NMD machinery further contributed to viral RNA degradation.

NbSMG7 contains an ATG8-interacting motif and undergoes autophagy-dependent degradation. Interference with the NbSMG7-ATG8 interaction reduced NbSMG7 degradation and altered NMD-mediated antiviral activity, demonstrating that autophagy forms a negative-feedback mechanism that compromises NMD-dependent restriction of CGMMV [48]. Nevertheless, this mechanism has not been functionally demonstrated in cucumber, and there is currently no direct evidence that autophagy selectively degrades CGMMV replication proteins.

## 3. Genetic and Genomic Basis of CGMMV Resistance in Cucumber and Related Cucurbits

### 3.1. Resistance Resources and Inheritance Patterns

The genetic basis of resistance to CGMMV varies substantially among cucurbit species and cannot be explained by a single conserved inheritance model. In cucumber, strong resistance resources remain relatively rare, and the genetic architecture of resistance has only recently begun to be investigated using large diversity panels. A cucumber diversity panel comprising 226 elite lines, 40 landraces, and 23 hybrids was assembled for resistance evaluation against six viruses, although the number of accessions successfully phenotyped and genotyped differed among individual virus experiments. For CGMMV, the experimental broad-sense heritability was estimated at 0.93, indicating high repeatability of the phenotyping procedure but not demonstrating monogenic inheritance. Complete resistance to CGMMV was detected only in Asian landraces belonging to one genetic group, whereas most elite materials showed intermediate or susceptible phenotypes. The resistance distribution and the proportion of unexplained genetic variance suggested a predominantly polygenic architecture in cucumber [39]. The recent strain-diversity study further showed that cucumber resistance can differ between CGMMV lineages, supporting multi-isolate validation of resistance phenotypes [31].

Evidence from melon further demonstrates the diversity of CGMMV resistance inheritance. Rajamony et al. reported that resistance in symptomless carrier-type muskmelon accessions was governed by recessive polygenes rather than by a single major gene [82]. In the Korean melon accession ‘Chang Bougi’, resistance to the SH isolate of CGMMV was controlled by two complementary recessive genetic factors, designated *cgmmv-1* and *cgmmv-2* [47].

In watermelon, resistance derived from PI595203 is recessively associated but cannot be fully explained by a simple one-gene model. Genetic mapping and marker validation were performed in watermelon, whereas the mechanistic localization, MP-interaction, protoplast, and whole-plant loss-of-function assays were conducted in heterologous systems, including *Nicotiana benthamiana* [43]. WPRb therefore provides a strong cross-species model for a movement-related host factor, but not direct functional evidence for the cucumber ortholog.

Collectively, available studies indicate that CGMMV resistance is genetically heterogeneous across cucurbit crops. Cucumber resistance appears mainly quantitative and polygenic, melon resistance may involve recessive polygenes or two complementary recessive genetic factors, and watermelon resistance involves recessive loci including WPRb. Resistance expression may also depend on the host genotype, virus isolate, environmental conditions, and phenotyping stage. Consequently, resistance mechanisms identified in melon or watermelon should be treated as comparative references rather than directly extrapolated to cucumber.The reported resistance resources and inheritance patterns are summarized in Table 4.

**Table 4:** CGMMV resistance resources and inheritance patterns in cucurbit crops.

Species or Material	Virus Isolate or Condition	Resistance Phenotype and Inheritance	Current Breeding Interpretation	Key Literature (Journal)
Cucumber diversity panel and landraces	CGMMV; multi-accession evaluation	High broad-sense heritability; complete or strong resistance concentrated in Asian landraces; response appears quantitative	Useful cucumber donor pool and mapping resource; not evidence for a universal monogenic gene	Monnot et al. (2022), Horticulture Research [39]
Cucumber accessions	European and Asian CGMMV strains	Most accessions susceptible or partially resistant; responses differ by strain	Resistance is isolate-sensitive and requires standardized multi-isolate phenotyping	Crespo et al. (2018), Euphytica [38]
Muskmelon symptomless carriers	CGMMV	Resistance reported as recessive polygenic inheritance	Comparative donor concept; causal loci remain unresolved	Rajamony et al. (1990), Euphytica [82]
Melon ‘Chang Bougi’	CGMMV-SH	Two complementary recessive genetic factors, *cgmmv-1* and *cgmmv-2*	Genetic factors rather than cloned genes; physical positions and causal genes unresolved	Sugiyama et al. (2007), Journal of the Japanese Society for Horticultural Science [47]
Melon and wild *Cucumis* materials	CGMMV isolates with differing virulence	Wild *Cucumis* species showed resistance in screening. In melon, ‘Chang Bougi’ showed temperature- and isolate-dependent resistance associated with restricted virus movement.	Potential comparative donors; cross-compatibility and transferability to cucumber require testing	Ruiz et al. (2021), Plants [91]; Sugiyama et al. (2006), Journal of the Japanese Society for Horticultural Science [92]
Watermelon PI595203	CGMMV	Recessive resistance associated with reduced cell-to-cell movement	Relevant comparative host-factor model, not direct cucumber evidence	Cai et al. (2023), Plant Physiology [43]
Watermelon *WPRb* allele	CGMMV	Watermelon G1282A/V428I variation was associated with resistance; marker validated in a backcross population; heterologous assays linked WPRb to movement restriction	Watermelon marker evidence plus heterologous functional evidence; cucumber ortholog remains unvalidated	Cai et al. (2023), Plant Physiology [43]
EMS-mutagenized bottle gourd	CGMMV; greenhouse and field screening	Asymptomatic and ELISA-negative M3 plants were identified; four lines retained field resistance	Promising mutagenized resource; inheritance and causal mutation require confirmation	AL-Kubati et al. (2022), Australasian Plant Pathology [93]

### 3.2. Mapping and Functional Validation of Resistance-Associated Loci

Compared with melon and watermelon, relatively few CGMMV resistance loci have been mapped in cucumber. The most reliable current evidence was obtained through a multilocus genome-wide association study. Two loci associated with cucumber response to CGMMV were identified. The chromosome 5 interval, designated QTLCgmmvCH5, spanned approximately 1.2 Mb, and its top-associated SNP explained approximately 24% of the phenotypic variance. QTLCgmmvCH2 on chromosome 2 covered approximately 5.8 kb, and its top-associated SNP explained approximately 5% of the phenotypic variance. Because the chromosome 2 association only marginally exceeded the significance threshold and had a minor allele frequency of 0.07, it requires validation in independent populations [39].

The chromosome 5 locus was located within a broader resistance hotspot containing QTLs associated with resistance to CGMMV, Cucumber mosaic virus, Cucumber vein yellowing virus, and Watermelon mosaic virus. The top CGMMV-associated SNP within the chromosome 5 hotspot was located in the coding region of a gene annotated as WRKY transcription factor 55, while two RDR1 genes were located nearby. However, WRKY55 and the neighboring RDR1 genes have not been functionally established as causal determinants of CGMMV resistance [39]. The *Arabidopsis* WRKY30-Cucumber mosaic virus study provides comparative support for prioritizing WRKY-family candidates, but it does not establish WRKY55 as a CGMMV-resistance gene in cucumber [94]. A schematic view of the cucumber loci and the comparative WPRb evidence is provided in Figure 4.

The strongest functional evidence for a CGMMV resistance-associated host factor in cucurbits currently comes from the watermelon WPRb study. The G1282A/V428I variant was associated with recessive resistance in watermelon, and an allele-specific marker was validated in a watermelon backcross population. Subcellular localization, MP-interaction, protoplast, and whole-plant functional assays were performed in heterologous systems, including *Nicotiana benthamiana*, and support a role for WPRb in plasmodesmata-mediated movement [43]. This provides a comparative model for investigating movement-related susceptibility factors in cucumber, but the cucumber WPRb ortholog has not yet been functionally validated.

Future mapping studies should combine standardized multi-isolate phenotyping, larger biparental or natural populations, fine mapping, and functional validation to distinguish causal genes from linked molecular markers.The mapped loci, candidate genes, and molecular markers relevant to CGMMV resistance are summarized in Table 5.

**Table 5:** CGMMV resistance-associated loci, candidate genes, and molecular markers.

Species	Locus or Gene	Evidence and Reported Estimate	Validation Status	Breeding Use	Key Literature (Journal)
Cucumber	QTLCgmmvCH5, chromosome 5	Approx. 1.2-Mb interval; top SNP explained approx. 24% of phenotypic variance; *WRKY55* annotation and nearby *RDR1* genes	GWAS association only; causal gene unconfirmed	Candidate marker after independent and multi-isolate validation	Monnot et al. (2022), Horticulture Research [39]
Cucumber	QTLCgmmvCH2, chromosome 2	Approx. 5.8-kb interval; top SNP explained approx. 5%; minor allele frequency 0.07	Marginal association; independent validation required	Research marker only until replicated	Monnot et al. (2022), Horticulture Research [39]
Cucumber	*CsRDR1c1/c2*	Suppression increased viral accumulation, supporting antiviral function	Functional contribution reported; natural allele-resistance relationship unresolved	Candidate defense target, not yet a diagnostic resistance marker	Leibman et al. (2018), Molecular Plant Pathology [40]
Watermelon	*ClWPRb*, G1282A/V428I	Watermelon genetic mapping/backcross marker validation; heterologous plasmodesmata localization, MP interaction, protoplast, and whole-plant functional assays	Strong cross-system evidence for a watermelon-associated factor; not validated in cucumber	Watermelon allele tracking; comparative cucumber target prioritization	Cai et al. (2023), Plant Physiology [43]
Melon	*cgmmv-1* and *cgmmv-2*	Segregation analysis supports two complementary recessive genetic factors	Physical positions and causal genes unresolved; not cloned	Donor tracking after revalidation; avoid gene-level claims	Sugiyama et al. (2007), Journal of the Japanese Society for Horticultural Science [47]
*Arabidopsis* (comparative)	WRKY30	WRKY30 mediates resistance to Cucumber mosaic virus	Comparative virus evidence; does not validate cucumber *WRKY55* for CGMMV	Candidate-prioritization rationale only	Zou et al. (2019), Biochemical and Biophysical Research Communications [94]

### 3.3. Genomic Resources Supporting CGMMV Resistance Gene Discovery

The development of cucumber genomic resources has substantially improved the technical foundation for resistance-gene discovery. Earlier cucumber genetic maps were limited by low marker density and inconsistent linkage-group assignments. Ren et al. constructed an integrated genetic and cytogenetic map containing 995 simple sequence repeat markers distributed across seven linkage groups. The map spanned 573 cM, with an average marker interval of approximately 0.58 cM, and was anchored to the seven cucumber chromosomes using fluorescence in situ hybridization [95].

Whole-genome resequencing subsequently shifted cucumber genetics from marker-limited linkage analysis toward population-scale genomic analysis. Qi et al. generated a genomic variation map containing approximately 3.6 million variants by deeply resequencing 115 cucumber lines selected from 3342 accessions worldwide. This resource revealed substantial population structure, domestication-associated bottlenecks, and genome-wide nucleotide variation and provided the basis for subsequent GWAS and genomics-assisted breeding [96].

More recently, a graph-based cucumber pan-genome was constructed from 12 chromosome-scale genome assemblies. Approximately 4.3 million genetic variants, including 56,214 structural variants, were identified. The pan-genome improved the representation of insertions, deletions, inversions, copy-number variants, and other sequences that cannot be adequately captured using a single linear reference genome [97]. Although this resource has not yet directly identified a causal cucumber gene for CGMMV resistance, it provides an important platform for testing whether the chromosome 5 resistance hotspot or other CGMMV-associated regions contain structural variants missed by SNP-based GWAS. The cucurbit pan-genome literature provides a broader rationale for incorporating structural variation into resistance-gene discovery [98].

These advances indicate that future cucumber CGMMV research should move beyond conventional linkage mapping alone. A robust discovery pipeline should integrate standardized phenotyping using multiple genetically characterized CGMMV isolates, whole-genome resequencing, structural-variation-aware association analysis, transcriptomic profiling, and functional validation through gene editing or complementation assays. Because cucumber resistance appears polygenic, genomic selection may also be more suitable than single-marker selection when multiple small-effect loci contribute to resistance. The existing chromosome 5 and chromosome 2 associations represent useful starting points, but neither locus should be treated as a fully validated breeding target until its effect is replicated in independent genetic backgrounds and field-relevant environments. The original GWAS authors similarly concluded that additional QTLs remain to be identified and suggested genome-wide selection as a possible strategy for complex CGMMV resistance [39]. General genomic-selection studies support the use of genome-wide marker information for complex traits, while its value for CGMMV resistance still depends on cucumber-specific training populations and phenotyping [99,100,101,102,103].

## 4. Advanced Breeding Technologies for CGMMV Resistance in Cucumber

### 4.1. Conventional Breeding and Resistance Resource Utilization

Conventional breeding remains a fundamental approach for incorporating CGMMV resistance into elite cucumber backgrounds. In principle, resistant donor accessions are crossed with agronomically superior but susceptible cultivars, followed by repeated selection and, where necessary, backcrossing to recover desirable yield, fruit quality, and adaptation traits. However, the practical application of this strategy in cucumber is currently constrained by the scarcity of well-characterized resistance donors and the incomplete understanding of the inheritance and isolate specificity of available resistance. Therefore, conventional breeding programs should not rely solely on symptom-based selection under natural infection. Candidate materials should be evaluated using standardized artificial inoculation, quantitative assessment of viral accumulation, multiple CGMMV isolates, and repeated tests across developmental stages and environments [10,38,91]. Recent CGMMV fruit and production studies further support evaluating both viral accumulation and market-relevant fruit traits during resistance assessment [16,17].

Because available evidence indicates that cucumber resistance to CGMMV is frequently quantitative rather than controlled by a universally effective major gene, phenotypic selection should be conducted across multiple generations and combined with molecular information whenever validated loci or markers are available [39]. Backcrossing may be used to recover elite genetic backgrounds, but resistance stability, possible linkage drag, and agronomic performance must be assessed before a donor can be considered suitable for cultivar development. Thus, conventional breeding should be regarded as the phenotypic and genetic foundation of CGMMV resistance improvement rather than as an independently sufficient solution.

#### 4.1.1. Screening and Identification of Resistance Resources

The identification of reliable resistance donors is the first and most critical step in conventional breeding for CGMMV resistance. Screening should distinguish true resistance from tolerance, delayed symptom development, and symptomless infection. Accordingly, resistance evaluation should combine visual disease scoring with molecular or serological quantification of viral accumulation in inoculated and systemic tissues. Because CGMMV resistance can be isolate-specific and affected by environmental conditions, candidate accessions should also be evaluated using genetically distinct virus isolates and under controlled temperature conditions before being introduced into breeding populations [47,91]. Detection studies using RT-qPCR, RT-LAMP, pre-amplification RT-qPCR, droplet digital PCR, and serology provide complementary tools for confirming infection status and viral accumulation [23,104,105,106,107,108].

In cucumber, screening of *Cucumis sativus* germplasm has shown that most accessions are susceptible to CGMMV, whereas only a limited number exhibit partial resistance or reduced viral accumulation [38]. A subsequent genome-wide association study identified complete or strong resistance primarily within a restricted group of Asian landraces, further indicating that useful cucumber resistance resources are uncommon and unevenly distributed among genetic groups [39]. These cucumber accessions are more directly relevant to cucumber breeding than resistance identified in other cucurbit species, but their resistance must still be validated against multiple CGMMV isolates and in independent genetic backgrounds before they are used as stable donor parents.

In watermelon, WPRb was identified as a resistance-associated host factor through watermelon genetic mapping and marker validation; complementary heterologous functional assays linked WPRb to plasmodesmata-mediated CGMMV movement [43]. This finding provides a valuable mechanistic reference for identifying homologous susceptibility factors in cucumber, but WPRb-associated watermelon germplasm cannot be described as a directly transferable cucumber donor without evidence from compatible crossing or functional validation of the cucumber ortholog.

A CGMMV-resistant wild watermelon accession has also been used to investigate the contribution of salicylic acid and flavonoid metabolism to resistance. The resistant accession exhibited higher basal levels of SA- and flavonoid-associated gene expression and metabolites than a susceptible cultivar, and flavonoid-containing extracts reduced CGMMV accumulation [89]. These findings enhance the mechanistic value of the germplasm, but transcriptomic or metabolic differences alone do not establish its suitability as a breeding donor or prove that the corresponding traits can be transferred to cucumber.

In melon, the accession ‘Chang Bougi’ restricts the systemic movement of the CGMMV-SH isolate, although symptom expression is temperature-dependent. The accession remains susceptible to the watermelon isolate CGMMV-W and to Kyuri green mottle mosaic virus, demonstrating that its resistance is isolate-specific rather than broadly effective against cucurbit-infecting tobamoviruses. Subsequent genetic analysis showed that resistance to CGMMV-SH is controlled by two complementary recessive genetic factors, designated *cgmmv-1* and *cgmmv-2* [47].

Wild *Cucumis* species may provide additional sources of CGMMV resistance. In a multi-isolate screening study, *C. anguria* and *C. ficifolius* remained symptomless and showed no detectable virus accumulation, whereas responses in *C. myriocarpus* and several *C. melo* accessions were isolate-dependent [91]. Nevertheless, these materials cannot yet be regarded as directly transferable cucumber donors because their cross-compatibility with *C. sativus*, hybrid fertility, resistance inheritance, and possible linkage drag require further evaluation [109]. At present, there is insufficient direct evidence to state that embryo-rescue-derived hybrids between wild and cultivated cucumber have produced validated CGMMV-resistant breeding materials. Consequently, such materials should be presented as potential resources for future exploration rather than as established achievements in CGMMV resistance breeding.

#### 4.1.2. Crossing, Backcrossing, and Selection for Resistance Introgression

Crossing and backcrossing provide a conventional framework for introgressing CGMMV resistance from validated cucumber donors into elite but susceptible genetic backgrounds. However, the breeding scheme must be designed according to the inheritance of the resistance source. For dominant major-gene resistance, resistant individuals may be identified directly in segregating generations. By contrast, when resistance is recessively inherited, F_1_ plants are generally heterozygous and may display a susceptible phenotype; therefore, apparently susceptible F_1_ plants should not be discarded solely on the basis of disease symptoms. Instead, resistance alleles should be retained through selfing, progeny testing, or marker-assisted genotyping when reliable markers are available. For quantitative resistance, which appears to be common in cucumber responses to CGMMV, repeated phenotypic selection across several generations and genetic backgrounds is required because individual loci may contribute only partial effects [39,110,111].

The direction of the initial cross should be determined by cross compatibility, seed production efficiency, maternal effects, and the agronomic characteristics of the parental lines, rather than by routinely assigning the resistant donor as the male parent. In each segregating or backcross generation, candidate plants should be evaluated using standardized mechanical inoculation and well-characterized CGMMV isolates. Resistance evaluation should combine symptom scoring with detection or quantification of viral accumulation in systemic tissues using RT-PCR, RT-qPCR, or ELISA. In a cucumber diversity-panel study, symptom severity was scored at approximately 14 days post-inoculation, whereas another germplasm-screening study evaluated symptom development and viral accumulation at later time points using both European and Asian CGMMV strains [39,91]. These differences indicate that inoculation conditions and evaluation times should be optimized for the plant material and virus isolate rather than applying a universal inoculum concentration or a fixed observation date.

Repeated backcrossing progressively increases the expected proportion of the recurrent-parent genome while retaining the resistance-associated donor region. Under the theoretical expectation of random segregation, BC_4_, BC_5_, and BC_6_ progenies contain approximately 96.88%, 98.44%, and 99.22% of the recurrent-parent genome, respectively. However, individual plants may deviate substantially from these expected values because of recombination and selection. Marker-assisted foreground selection can be used to retain the target resistance allele, whereas background selection can accelerate recovery of the recurrent-parent genome and reduce linkage drag [110,111].

In watermelon, an allele-specific marker based on the G1282A variation in WPRb was validated in a BC_1_ population, demonstrating marker-tracking utility within watermelon [43]. This marker validation should not be conflated with the development of commercial resistant lines through four to six generations of backcrossing, and it does not demonstrate that WPRb can be directly introgressed into cucumber. Therefore, the study should be presented as a watermelon breeding reference rather than as evidence of an established cucumber backcross-breeding program.

### 4.2. Molecular Breeding Technologies

#### 4.2.1. Molecular Marker-Assisted Selection: Current Evidence and Limitations

Molecular marker-assisted selection (MAS) is an important complement to conventional breeding because it enables resistance-associated alleles or genomic regions to be tracked independently of visible disease symptoms. Functional markers derived from causal sequence variants generally provide the highest selection reliability, whereas markers linked to quantitative trait loci may be affected by recombination and genetic background. MAS can therefore improve early-generation selection efficiency and assist foreground and background selection during backcrossing. However, it cannot fully replace artificial inoculation and phenotypic evaluation, particularly when resistance is quantitative, isolate-specific, or environmentally sensitive [111,112].

In cucumber, the currently available molecular evidence for CGMMV resistance is primarily based on genome-wide association analysis rather than validated functional markers. Two resistance-associated loci, QTL_CgmmvCH5_ and QTL_CgmmvCH2_, were identified on chromosomes 5 and 2, respectively [39]. The chromosome 5 locus showed a comparatively larger phenotypic effect, whereas the chromosome 2 association was weaker and occurred at a relatively low minor allele frequency. Although the associated SNPs provide useful starting points for marker development, they have not yet been validated as stable diagnostic markers in independent biparental populations or diverse cucumber genetic backgrounds. Therefore, these loci should currently be regarded as candidate marker resources rather than immediately applicable functional markers for cucumber breeding [39].

The clearest CGMMV-associated molecular marker currently available in cucurbits was developed from the watermelon WPRb locus. The G1282A substitution was associated with resistance, and an allele-specific marker was validated in a watermelon backcross population [43]. Thus, the marker has value for tracking the resistance-associated allele in watermelon, but it is not a validated cucumber marker. Before application to cucumber populations, the corresponding cucumber ortholog, allelic variation, and association with CGMMV response must be independently evaluated [43].

Functional studies in watermelon have identified several CGMMV-responsive host factors, including ClAGO1 and ClAGO5 in antiviral RNA silencing and ClGST in ROS-associated defense [44,65]. However, molecular markers derived from these genes have not been validated in segregating cucumber populations. These genes therefore provide candidate loci for future marker development but cannot currently be used as validated markers for MAS in cucumber. Similarly, the melon loci designated *cgmmv-1* and *cgmmv-2* have been defined genetically, but they are not cloned genes; their physical positions, causal sequences, and validated InDel markers remain unresolved [47].

Accordingly, the current application of MAS for CGMMV resistance should follow a cautious two-stage strategy. Resistance-associated markers may first be used to enrich candidate individuals or retain donor alleles during population development. These plants should subsequently undergo standardized artificial inoculation, quantitative detection of viral accumulation, and agronomic evaluation. Marker performance should also be tested across multiple cucumber genetic backgrounds and genetically distinct CGMMV isolates before routine breeding application. This integration of molecular preselection with phenotypic validation can reduce selection workload while avoiding false-positive selection caused by incomplete marker-trait linkage.

#### 4.2.2. Application of Molecular Design Breeding to CGMMV Resistance

Molecular design breeding seeks to connect genomic and multi-omics information with gene discovery, trait prediction, and breeding decision-making, increasingly supported by machine-learning and genomic-prediction approaches [113]. For CGMMV resistance in cucumber, however, molecular design breeding should currently be regarded as an emerging framework rather than a fully established breeding system. Available resources include CGMMV-associated loci identified through genome-wide association analysis, the cucumber genomic variation map, and the graph-based cucumber pan-genome [39,96,97]. The broader pangenomic context and genomic-selection literature support using structural variants and genome-wide prediction as complementary resources [98,99,100,101,102,103]. These resources provide a foundation for identifying SNPs, structural variants, and candidate genes associated with resistance, but the causal genes underlying cucumber resistance to CGMMV have not yet been conclusively validated.

A practical molecular design breeding pipeline for CGMMV resistance should begin with standardized phenotyping using genetically characterized virus isolates and cucumber populations representing broad genetic diversity [38,39]. Linkage mapping, genome-wide association studies, whole-genome variation analysis, and structural-variation-aware genomic analysis can subsequently be used to identify resistance-associated regions and candidate alleles [39,95,96,97]. Candidate genes within these regions should be prioritized by integrating transcriptomic, metabolomic, and host–virus protein-interaction evidence [43,44,65]. Functional validation through gene silencing, overexpression, protein-interaction assays, or genome editing is then required to distinguish causal resistance determinants from genes that merely respond to CGMMV infection [43,44,65]. RNA-based design may provide an additional route by prioritizing conserved viral sequences as silencing targets; polycistronic amiRNAs directed against the CGMMV replicase, movement protein, and coat protein have conferred enhanced resistance in transgenic cucumber [41].

Once reliable resistance loci or functional alleles have been established, marker-assisted selection may be suitable for major-effect loci, whereas genomic selection may be more appropriate when resistance is controlled by numerous small-effect loci [111,112,114]. Because molecular-design and prediction models depend on relationships among genotypes, phenotypes, and environments, molecular information should remain coupled with phenotypic and agronomic evaluation [113]. For CGMMV resistance breeding, candidate materials must additionally be evaluated for yield, fruit quality, fertility, plant vigor, and environmental adaptability. Therefore, molecular design breeding should not replace conventional phenotypic selection; rather, it should establish an integrated pipeline connecting resistance-resource evaluation, genomic discovery, functional validation, molecular selection, and cultivar development [113].

#### 4.2.3. Genome Editing and RNA-Based Resistance Engineering

Genome editing and RNA-based engineering provide complementary strategies for developing CGMMV-resistant cucumber germplasm. CRISPR/Cas9-mediated editing of cucumber *eIF4E* has demonstrated the feasibility of generating resistance to several eIF4E-dependent viruses, although resistance to CGMMV was not established in that study [34]. General plant genome-editing reviews describe the technical scope of CRISPR/Cas systems, whereas no cucumber editing target has yet been functionally validated for CGMMV [36,37,115]. For WPRb, the resistance-associated allele was identified in watermelon, and the molecular/functional evidence was obtained through watermelon genetic analysis and heterologous assays, including *N. benthamiana* [43]. The corresponding cucumber ortholog therefore remains a candidate target requiring independent validation.

Other CGMMV-responsive genes, including *ClAGO1*, *ClAGO5*, and *ClGST*, have been functionally investigated in watermelon, but their corresponding cucumber homologs remain candidates rather than validated editing targets for CGMMV resistance [44,65]. By contrast, stable transgenic cucumber lines expressing polycistronic artificial microRNAs directed against conserved regions of the CGMMV replicase, movement protein, and coat protein showed reduced viral accumulation and enhanced resistance [41]. Comparable CGMMV-targeted RNA-silencing constructs have also been evaluated in *N. benthamiana*, melon, and watermelon, providing comparative rather than cucumber-specific evidence [116,117,118]. However, strain-dependent effects were observed, and the durability of this strategy against genetically diverse CGMMV isolates and under production conditions remains to be established. The newly described rORF1/rORF2 coding regions are relevant to CGMMV virulence, but the study supporting their characterization did not establish an RNA-based resistance strategy; their conservation, target accessibility, and resistance efficacy therefore remain unknown [57]. Exogenous dsRNA has also reduced CGMMV infection in watermelon, but this result remains a related-cucurbit proof of concept rather than evidence of a stable cucumber breeding trait [119]. General amiRNA studies further show that target accessibility, viral sequence variation, and escape evolution must be considered when durability is assessed [120,121,122,123,124,125]. Therefore, cucumber-specific host factors must be functionally validated and assessed for pleiotropic effects before genome editing is applied.

#### 4.2.4. Precision Parental Selection and Resistance Pyramiding

Precision parental selection should integrate resistance phenotype, resistance-associated genotype, genetic diversity, and agronomic performance. The CGMMV-associated loci identified on cucumber chromosomes 5 and 2 may assist parental selection, but they still require validation in independent populations and against multiple virus isolates [39]. Major-effect resistance alleles may be tracked through MAS, whereas genomic selection may be more suitable for polygenic resistance [111,112,114].

Resistance pyramiding aims to combine mechanisms acting at different stages of infection. Natural quantitative resistance, validated susceptibility-factor alleles, and RNA-based resistance may potentially be combined through crossing and molecular selection. However, such combinations have not yet been demonstrated for CGMMV resistance in cucumber and should therefore be presented as a future strategy rather than an established breeding system [39,41,43].

#### 4.2.5. Proposed Integrated Breeding Framework

An integrated framework combining conventional crossing, molecular selection, and precision engineering may accelerate CGMMV resistance improvement in cucumber. The proposed pipeline includes four stages: resistance-resource screening using standardized inoculation [39,91], genomic identification of resistance-associated loci and structural variants [39,96,97], functional validation or editing of candidate genes [38,43,44], and introgression of validated resistance components into elite cucumber backgrounds through crossing and molecular selection [111,112]. General breeding studies support combining major-effect-marker selection with genome-wide prediction for complex resistance, but each component still requires CGMMV-specific validation [99,100,101,102,103,114]. The relative breeding-stage sequence and decision gates are summarized in Figure 5.

Advanced lines should subsequently be evaluated for resistance against multiple CGMMV isolates, as well as for yield, fruit quality, fertility, and environmental adaptability. Implementation of this proposed framework in cucumber breeding requires validation of its component strategies under multi-isolate and multi-environment conditions. The main advantages, limitations, and research priorities of these strategies are summarized in Table 6.

## 5. Conclusions and Perspectives

Considerable progress has been made in understanding CGMMV infection and resistance in cucumber and related cucurbit crops. Host–virus protein interactions, RNA silencing, phytohormone signaling, reactive oxygen species regulation, and RNA or protein quality-control pathways have provided important mechanistic insights. However, direct functional evidence in cucumber remains limited, because several key mechanisms, including WPRb-associated movement restriction, AGO-associated defense, and ROS-associated responses, have been supported mainly by watermelon studies and heterologous *Nicotiana benthamiana* assays rather than by cucumber validation [43,44,65]. Recent studies of cucumber fatty-acid metabolism and related cucurbit chlorophyll responses expand the candidate mechanism set, but they do not remove the need for cucumber-specific genetic validation [42,66]. In cucumber, genome-wide association analysis has identified CGMMV-associated regions on chromosomes 5 and 2, but their causal genes have not yet been functionally confirmed [39]. By contrast, polycistronic artificial microRNAs targeting conserved CGMMV genes have provided direct experimental evidence for RNA-based resistance in cucumber [41]. The available evidence therefore supports a transition from descriptive mechanism studies toward cucumber-specific genetic validation and breeding translation.

First, future research should prioritize the identification and validation of cucumber-specific resistance determinants. Standardized phenotyping using multiple genetically characterized CGMMV isolates should be combined with linkage mapping, genome-wide association studies, whole-genome resequencing, transcriptomics, and structural-variation-aware pan-genome analysis [39,91,96,97]. Candidate genes must subsequently be verified through gene silencing, complementation, protein-interaction assays, or genome editing before they are described as resistance genes or used as breeding targets. The available cucumber genomic variation map and graph-based pan-genome provide valuable resources for this purpose [96,97].

Second, host-factor editing should be pursued only after direct functional validation. Editing cucumber *eIF4E* demonstrates the technical feasibility of generating resistance to several eIF4E-dependent viruses, but it does not establish eIF4E as a CGMMV susceptibility factor [34]. WPRb provides a relevant movement-related model: its resistance association was established in watermelon, while supporting localization, interaction, and loss-of-function evidence came from heterologous assays [43]. Nevertheless, the cucumber WPRb ortholog must be characterized independently, and possible effects on development, yield, and fruit quality must be evaluated before stable edited materials are developed.

Third, resistance improvement should combine complementary mechanisms rather than depend on a single candidate gene. Natural quantitative resistance, validated host-factor alleles, and RNA-based resistance may potentially be integrated through crossing, molecular selection, and precision engineering [39,41]. However, resistance pyramiding should be performed only after each component has been independently validated. Particular attention should be given to isolate specificity, viral sequence variation, possible escape mutants, epistatic interactions, and agronomic fitness. Artificial microRNA technology has already produced promising CGMMV-resistant cucumber materials, but its durability across diverse virus populations and production environments remains to be determined [41].

Finally, an integrated breeding pipeline should connect resistance-resource screening, genomic discovery, functional validation, molecular selection, and multi-environment evaluation. Marker-assisted selection may be effective for validated major-effect alleles, whereas genomic selection may be more appropriate for quantitative resistance controlled by multiple loci. Advanced materials should be evaluated not only for symptom suppression and reduced viral accumulation, but also for yield, fruit quality, fertility, genetic stability, and resistance across multiple CGMMV isolates. The practical value of this integrated pipeline remains to be established through genetic validation, multi-isolate resistance testing, and multi-environment agronomic evaluation.

In conclusion, durable CGMMV resistance in cucumber will depend on rigorous cucumber-specific validation rather than direct extrapolation from related cucurbit species or other virus–host systems. Integrating reliable phenotyping, genomic resources, functional genetics, molecular selection, and precise engineering offers a realistic route toward CGMMV-resistant cucumber cultivars. The central challenge is not the availability of breeding technologies, but the identification of causal resistance determinants that provide stable protection without compromising agronomic performance.

## Figures and Tables

**Figure 1:**
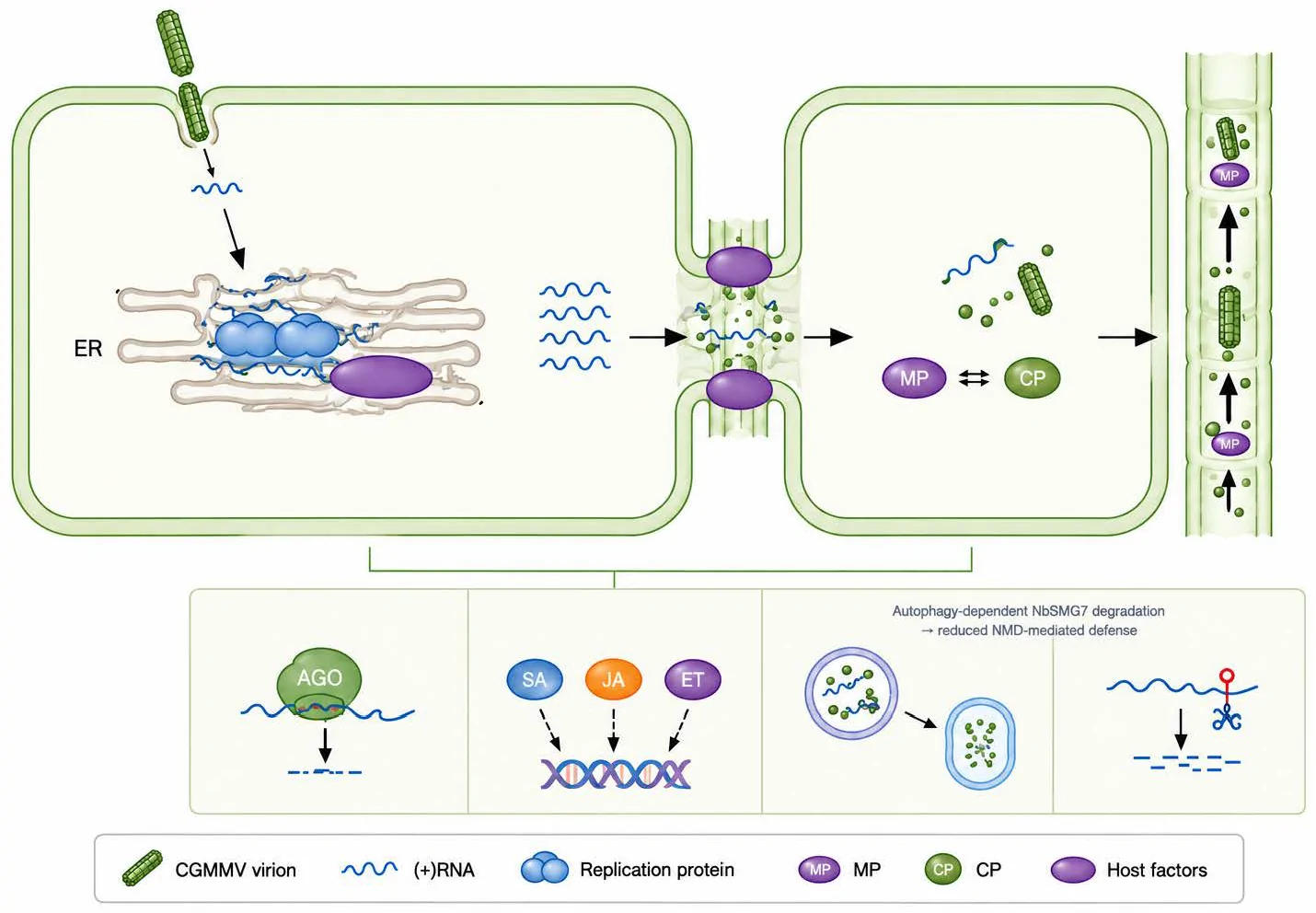
Proposed model of CGMMV infection and host defense mechanisms.

**Figure 2:**
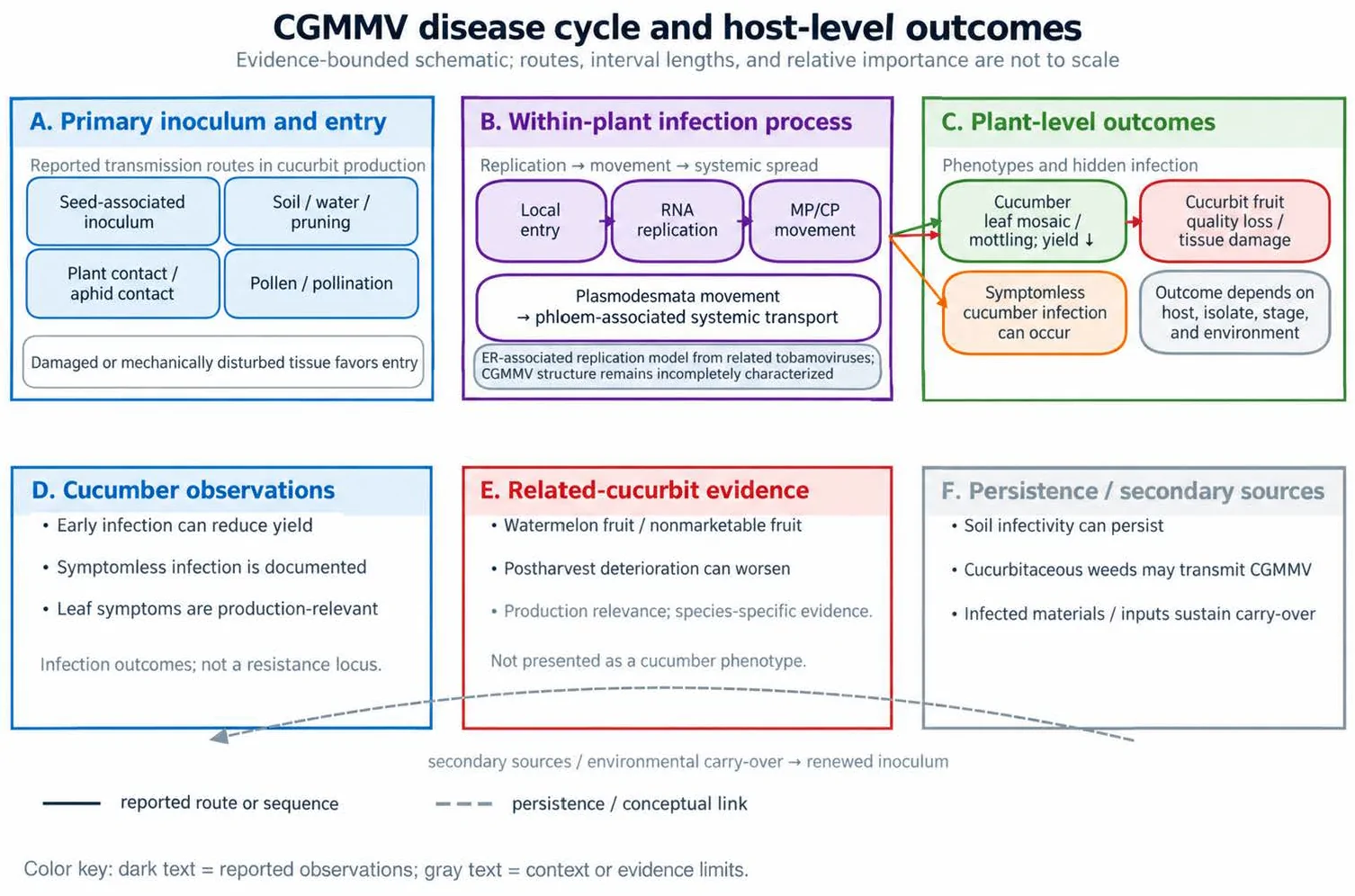
CGMMV disease cycle and host-level outcomes. The schematic illustrates seed-associated introduction, environmental persistence, contact and pollination transmission, within-plant movement, and disease symptoms in cucumber and related cucurbit crops. Cucumber-specific observations are distinguished from related-cucurbit evidence. The figure was redrawn based on the cited literature [2,3,4,5,6,7,8,9,11,12,13,14,15,16,17,54,55,60,61,62,63,64]. The scientific content was prepared and verified by the authors; OpenAI Codex (GPT-5) was used only for layout, vector drawing, and typesetting. The figure contains no original experimental data.

**Figure 4:**
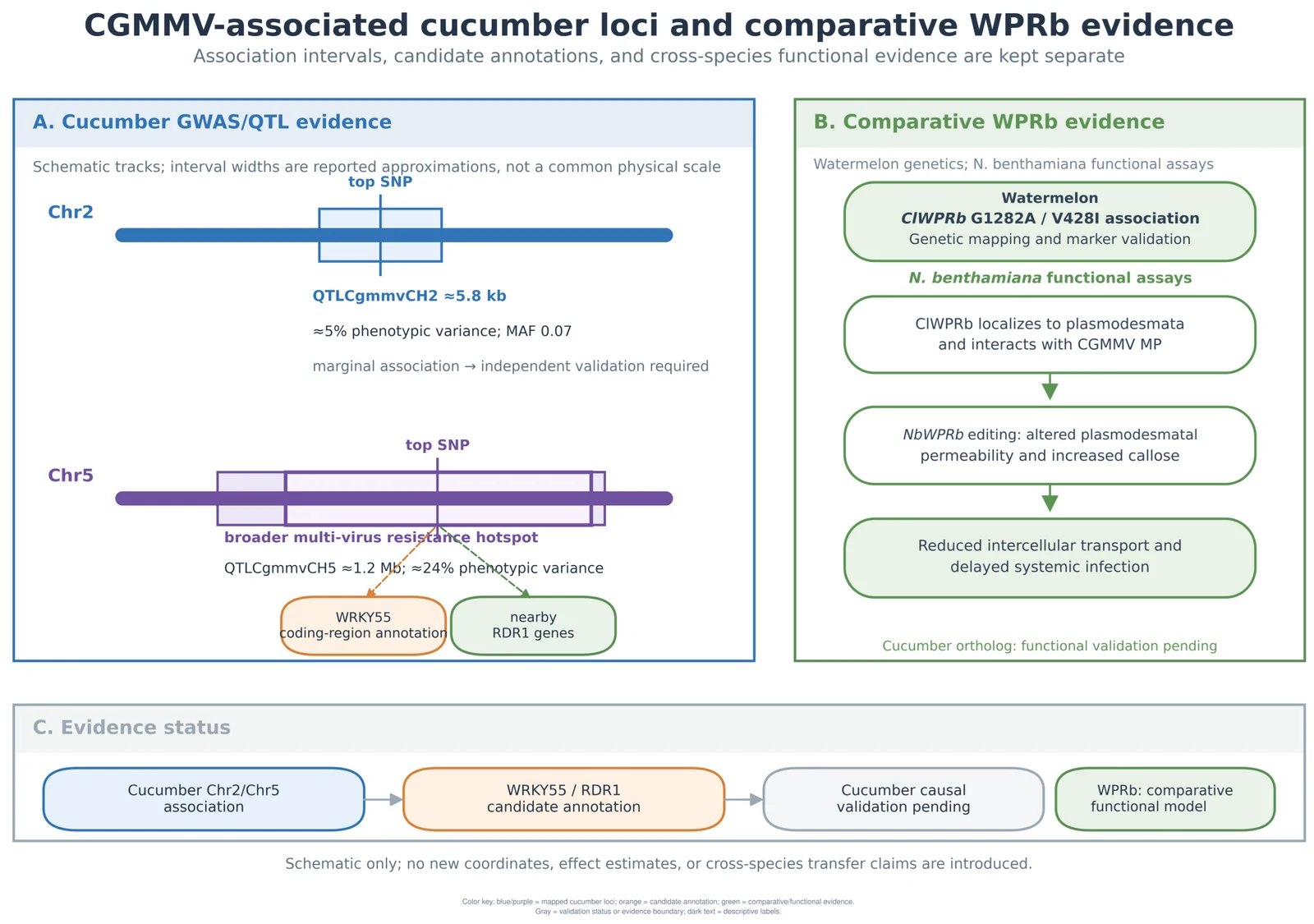
CGMMV-associated cucumber loci and comparative WPRb evidence. The schematic presents the cucumber loci QTLCgmmvCH2 and QTLCgmmvCH5, together with their approximate intervals, phenotypic effects, and current validation status. *WRKY55* and neighboring *RDR1* genes are shown as candidate annotations rather than confirmed causal genes [39]. The watermelon panel summarizes the *ClWPRb* G1282A/V428I association and related functional evidence from watermelon and heterologous assays in *Nicotiana benthamiana* [43]. The figure distinguishes cucumber genetic associations from comparative watermelon evidence and does not imply that WPRb is a validated cucumber resistance gene. The figure was redrawn based on the cited literature [39,43]. The scientific content was prepared and verified by the authors; OpenAI Codex (GPT-5) was used only for layout, vector drawing, and typesetting. The figure contains no original experimental data.

**Figure 5:**
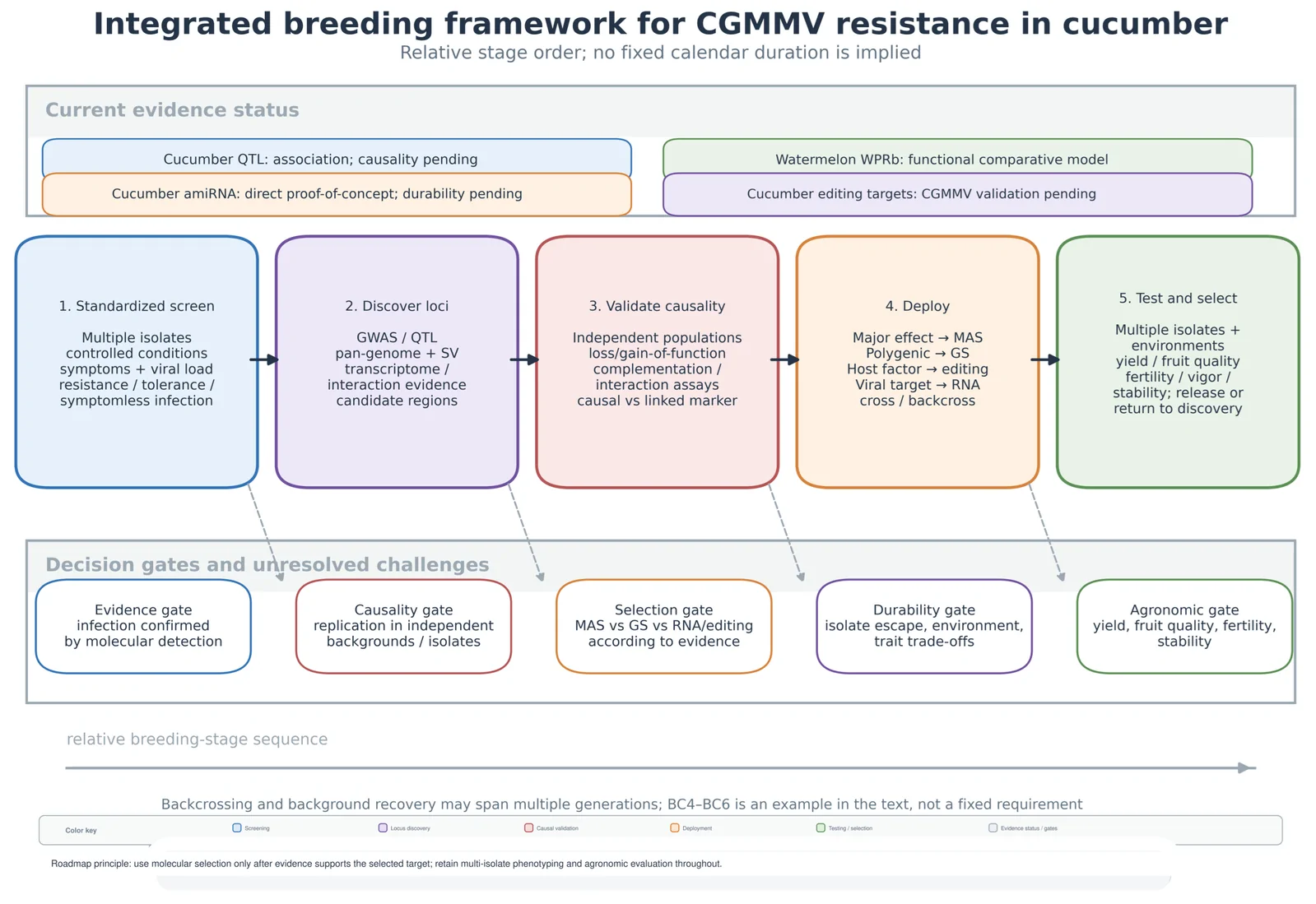
Integrated breeding framework for CGMMV resistance in cucumber. The schematic outlines a research workflow from standardized multi-isolate phenotyping to genomic discovery, functional validation, evidence-based deployment, and multi-environment agronomic evaluation. The framework is informed by cucumber and related-cucurbit CGMMV studies and general breeding literature [39,41,43,91,99,100,101,102,103,111,112,114]. The figure was redrawn based on the cited literature. The scientific content was prepared and verified by the authors; OpenAI Codex (GPT-5) was used only for layout, vector drawing, and typesetting. The figure contains no original experimental data.

**Table 1:** Evidence hierarchy and interpretation rules used in this review.

Evidence Category	Host System	Typical Observations	What the Evidence Supports	Key Literature (Journal)
Direct cucumber phenotyping and genetics	Cucumber (*Cucumis sativus*)	Standardized inoculation, disease score, viral load, heritability, GWAS/QTL	Cucumber-specific phenotype or association; not a causal gene without functional validation	Crespo et al. (2018), Euphytica [38]; Monnot et al. (2022), Horticulture Research [39]
Cucumber functional evidence	Cucumber (*Cucumis sativus*)	Perturbation, transgenic/amiRNA lines, expression or viral-load phenotyping	Mechanistic support in cucumber when perturbation and infection phenotype are concordant	Leibman et al. (2018), Molecular Plant Pathology [40]; Miao et al. (2021), International Journal of Molecular Sciences [41]; Jiang et al. (2025), Plant Cell Physiology [42]
Related-cucurbit comparative evidence	Watermelon, melon, bottle gourd	Host-factor, hormone, metabolic, or inheritance studies in other cucurbits	Comparative mechanism or donor hypothesis; not direct validation of a cucumber gene	Cai et al. (2023), Plant Physiology [43]; Liu et al. (2023), Molecular Plant Pathology [44]; Liu et al. (2024), Frontiers in Plant Science [45]; Li et al. (2023), International Journal of Molecular Sciences [46]; Sugiyama et al. (2007), Journal of the Japanese Society for Horticultural Science [47]
Model-plant and comparative tobamovirus evidence	*Nicotiana benthamiana*, tobacco, *Arabidopsis*; CGMMV or other tobamoviruses	Protein interactions, RNA-silencing pathways, or host susceptibility-factor studies	Hypothesis generation and target prioritization only; cucumber/CGMMV validation remains necessary	Chen et al. (2024), Advanced Science [48]; Asano et al. (2005), FEBS Letters [49]; Yamanaka et al. (2002), Journal of Virology [50]; Ishibashi and Ishikawa (2016), Annual Review of Phytopathology [51]
Functional-validation standard	Any host system	Perturbation or allele contrast plus infection phenotype/viral load; ideally complementation, independent backgrounds, or multiple isolates	Causal or near-causal interpretation; association alone remains a candidate signal	Garcia-Ruiz (2019), Molecular Plant Pathology [52]; Nicaise (2014), Frontiers in Plant Science [53]

**Table 6:** Evidence-Based Comparison of Major Strategies for CGMMV Resistance Improvement in Cucumber.

Strategy	Current Evidence	Main Advantage	Major Limitation	Research Priority	Key Supporting Literature (Journal)
Natural resistance and molecular selection	CGMMV-associated loci identified on cucumber chromosomes 5 and 2, but causal genes remain unconfirmed [39].	Uses natural variation and is readily compatible with conventional breeding	Quantitative effects, isolate specificity, and insufficient marker validation	Validate loci in independent populations and develop diagnostic markers	Monnot et al. (2022), Horticulture Research [39]; Crespo et al. (2018), Euphytica [38]
Host-factor genome editing	Cucumber *eIF4E* editing is effective against several other viruses; WPRb evidence combines watermelon genetic data with heterologous functional assays [34,43].	Can generate precise and potentially transgene-free resistant alleles	No validated cucumber host-editing target for CGMMV; possible pleiotropic effects	Identify and validate cucumber-specific susceptibility factors	Chandrasekaran et al. (2016), Molecular Plant Pathology [34]; Cai et al. (2023), Plant Physiology [43]
RNA-based resistance	Polycistronic amiRNAs have enhanced CGMMV resistance in transgenic cucumber [41].	Directly targets multiple essential viral genes	Sequence-dependent resistance, potential viral escape, and regulatory constraints	Test conserved targets against diverse CGMMV isolates	Miao et al. (2021), International Journal of Molecular Sciences [41]; Wang et al. (2025), Agronomy [119]
Integrated resistance pyramiding	Proposed framework combining natural resistance, molecular selection, and engineered resistance	May reduce dependence on a single resistance mechanism	No complete CGMMV pyramiding system has yet been validated in cucumber	Validate each component before combining mechanisms	Merrick et al. (2021), Frontiers in Plant Science [114]; Carr (2024), Viruses [115]

## Data Availability

No new data were created or analyzed in this study. Data sharing is not applicable.
